# Ligand specificity of Killer cell Immunoglobulin-like Receptors: a brief history of KIR

**DOI:** 10.3389/fimmu.2012.00394

**Published:** 2012-12-24

**Authors:** Jeroen van Bergen, John Trowsdale

**Affiliations:** ^1^Department of Immunohematology and Blood Transfusion, Leiden University Medical CenterLeiden, Netherlands; ^2^Immunology Division, Department of Pathology, Cambridge UniversityCambridge, UK

Natural Killer (NK) cells are a subset of innate lymphoid cells specialized for cytotoxicity and production of inflammatory cytokines such as IFNγ. As such, they are presumed to be important for early control of infections with intracellular pathogens and anti-tumor immunity. In addition, NK cells may help to regulate placentation. The activation of effector functions is controlled by the integration of signals from multiple receptors, including not only activating and inhibitory NK cell receptors but also receptors for cytokines such as IL-12, -15, -18. The Killer cell Immunoglobulin-like Receptors (KIR) make up the largest and by far most polymorphic human NK cell receptor family. Underscoring the importance of KIR, their genetic variation is associated with resistance to viruses such as HCV and HIV, auto-immune diseases and reproductive success. To understand how KIR influence health and reproduction, it is critical to know what molecules they interact with. In this topic, leading experts provide an up-to-date overview of the known ligand specificities of specific KIR.

Cresswell and deMars first showed in 1989 that transfection of HLA class I molecules into class I-deficient cell lines reduced their susceptibility to NK cell lysis (Harel-Bellan et al., 1986; Quillet et al., 1988; Shimizu and DeMars, 1989; Storkus et al., 1989). In this fashion, several HLA-B and -C alleles were identified as potential ligands for inhibitory NK cell receptors (Ciccone et al., 1992; Colonna et al., 1993; Cella et al., 1994; Mandelboim et al., 1996). Certain antibodies to NK cell surface molecules were able to restore lysis of these HLA class I transfectants by NK cell clones, allowing the definition of specificities of the receptors that were blocked by the antibodies. Most of the antibodies (GL183, EB6, CH-L, DX27, HP-3E4) bound 58 kDa receptors for HLA-C variants characterized by either an Asparagine (C1) or a Lysine (C2) at position 80 of their alpha-chains (Ciccone et al., 1992; Moretta et al., 1993; Mandelboim et al., 1996), others (DX9, Z27) a 70 kDa receptor for HLA-B (and some HLA-A) alleles carrying the “Bw4-motif” (Litwin et al., 1994; Gumperz et al., 1995), and a few (Q66, DX31) bound a 140 kDa homodimeric receptor for HLA-A3 and -A11 (Pende et al., 1996). In many individuals the antibodies recognizing inhibitory, 58 kDa receptors for HLA-C also bound 50 kDa proteins from NK cell clones that acted as activating receptors. Some of these receptors also bound HLA-C, although weakly (Moretta et al., 1995).

Molecular cloning revealed that these receptors carried 1–3 immunoglobulin-like extracellular domains that were very similar between different receptors (Colonna and Samaridis, 1995; D'Andrea et al., 1995; Wagtmann et al., 1995). The main differences between activating and inhibitory receptors were found in their cytoplasmic tails. While the inhibitory receptors had a long cytoplasmic tail with two ITIMs, allowing inhibitory signaling (Burshtyn et al., 1996; Olcese et al., 1996), the activating ones had a short cytoplasmic tail and a positively charged residue in their transmembrane domain, allowing association with the ITAM-bearing, signaling adapter DAP12 (Olcese et al., 1997; Lanier et al., 1998). As scientists generally would rather share toothbrushes than nomenclatures, for a while nomenclatures from different laboratories co-existed. However, since the receptors from different loci had remarkably high sequence similarity, in some cases over 97%, and clearly belonged to the same receptor family, it made sense to use a unified nomenclature that reflected the receptors' structures (Long et al., 1996). The receptors were named KIR. KIR2D (50, 58 kDa) and KIR3D (70, 140 kDa), refer to receptors with two or three IgSF domains, respectively. Furthermore, L stands for receptors having long and S for those having short cytoplasmic tails, consistent with the presence or absence of ITIM motifs, respectively. Each KIR subfamily is designated by an individual number, for example KIR2DS1 (Uhrberg et al., 1997; Valiante et al., 1997). The different KIR loci can be found, together with discontinued names, on the EBI-website: http://www.ebi.ac.uk/ipd/kir/genes.html (Robinson et al., 2010).

Molecular cloning of the KIR paved the way for more detailed genetic analyses. In the first instance, novel KIR were cloned from cDNA libraries by homology. In this way KIR2DL4 (Selvakumar et al., 1996), a receptor for HLA-G (Rajagopalan and Long, 1999; Rajagopalan et al., 2005), was identified. The KIR locus, located on chromosome 19q13.4, turned out to be densely packed with KIR genes, oriented in a head-to-tail fashion (Martin et al., 2000; Wilson et al., 2000). While some KIR haplotypes contained few KIR genes, others had many. These efforts also revealed the existence of additional KIR genes KIR2DL5A, KIR2DL5B, KIR2DS5, KIR3DL3 (Vilches et al., 2000; Gomez-Lozano et al., 2002), as well as several pseudogenes (KIR2DP1, KIR3DP1, KIR1D). Common to virtually all haplotypes is the presence of KIR3DL3 at the centromeric end of the locus, KIR2DL4 roughly in the center, and KIR3DL2 at the telomeric end. It also quickly became clear that individual KIR genes display extensive and functional polymorphism (O'Connor et al., 2007). The total number of functional KIR genes is 15, and well over 50 alleles for individual genes have been described (http://www.ebi.ac.uk/ipd/kir/alleles.html).

All this sequence information allowed the design of primer sets to rapidly determine the presence or absence of specific KIR genes (or alleles) in individuals (Uhrberg et al., 1997), which in turn allowed genetic association studies. The first report of this kind showed that the presence of KIR2DS2 was associated with vasculitis in rheumatoid arthritis patients (Yen et al., 2001; Majorczyk et al., 2007). Subsequent studies indicated a role for KIR in psoriatric arthritis (Martin et al., 2002b; Nelson et al., 2004; Williams et al., 2005), type I diabetes (van der Slik et al., 2003, 2007) and several other auto-immune conditions. In line with the notion that NK cells are important mediators of anti-viral immunity, variation in KIR genes associated with the ability to control infection with hepatitis C (Khakoo et al., 2004; Knapp et al., 2010; Dring et al., 2011) and HIV (Martin et al., 2002a, 2007). The largest studies of this kind showed epistatic interaction between KIR3DS1 and HLA-B in resistance to HIV progression (Martin et al., 2002a, 2007). Finally, interactions between maternal KIR and fetal HLA-C appeared to influence reproductive success (Hiby et al., 2004, 2010).

Even though these studies demonstrated an important role for KIR and KIR-HLA interactions in various diseases, the underlying mechanisms are only beginning to be understood (Anfossi et al., 2006; Alter et al., 2007, 2009; Ahlenstiel et al., 2008; Hiby et al., 2010; O'Connor et al., 2011; Tarek et al., 2012). A critical gap in our knowledge is the fact that ligands for many activating, and some inhibitory, KIR are unknown. Of the eight inhibitory KIR, five have well-characterized HLA specificities. Generally, KIR2DL1, KIR2DL2, KIR2DL3 together cover all HLA-C alleles, KIR3DL1 binds a subset of HLA-B alleles, and KIR3DL2 binds HLA-A3/A11 loaded with specific peptides as well as homodimers of HLA-B27 heavy chains. These binding profiles are supported in some cases by structural studies (Boyington et al., 2000; Fan et al., 2001; Campbell and Purdy, 2011; Vivian et al., 2011). Of the six activating KIR, only KIR2DS1 has a clearly defined specificity: C2 (Stewart et al., 2005). In this topic, leading experts discuss the state of the art in ligand identity for several KIR, in an effort to shed light on the contribution of KIR-ligand interactions to disease (Cisneros et al., 2012; Korner and Altfeld, 2012; Moesta and Parham, 2012; Rajagopalan and Long, 2012; Shaw and Kollnberger, 2012).
